# Comparison of short-term outcomes of robotic-assisted radical colon cancer surgery using the Kangduo Surgical Robotic System and the Da Vinci Si Robotic System: a prospective cohort study

**DOI:** 10.1097/JS9.0000000000000976

**Published:** 2023-12-04

**Authors:** Yunxiao Liu, Yuliuming Wang, Chunlin Wang, Xin Wang, Xin Zhang, Yihaoran Yang, Zhengqiang Wei, Yi Xiao, Guiyu Wang

**Affiliations:** a Department of Colorectal Cancer Surgery, The Second Affiliated Hospital of Harbin Medical University, Harbin; b Department of Gastrointestinal Surgery, The First Affiliated Hospital of Chongqing Medical University, Chongqing; c Department of General Surgery, Division of Colorectal Surgery, Peking Union Medical College, Hospital, Chinese Academy of Medical Sciences and Peking Union Medical College, Beijing, People’s Republic of China

**Keywords:** colon cancer, da Vinci Robotic System, Kangduo Robotic system, short-term outcomes

## Abstract

**Background:**

Robotic surgery has been a revolution for colon cancer (CC) patients, with the increasing availability of different competitive robotic systems, but evidence of relevant oncologic outcomes is indeed scarce. Our goal was to compare the surgical quality and short-term oncologic outcomes of the Kangduo Surgical Robotic System and the da Vinci Si Robotic System in patients with CC.

**Methods:**

These are results from a subcohort of a multicenter randomized controlled noninferiority trial performed in three centers in China. Enrolled patients were randomly assigned to undergo surgery using either the KD-SR-01 system (KD group) or the da Vinci Si (DV) robotic system (DV group). Neither investigators nor patients were masked to treatment allocation, but assessment of pathological outcomes was masked to treatment allocation. The primary endpoint was surgical success rate. The secondary endpoints were surgical outcomes, pathologic outcomes, and postoperative outcomes. The study is registered at www.chictr.org.cn (ChiCTR2200063172). Although the long-term follow-up results were not a predefined endpoint for this study, late-stage work is in progress.

**Results:**

A total of 58 CC patients were included in this study, 28 in the KD group and 30 in the DV group. All patients were successfully operated without any intermediate open/conventional laparoscopic surgery and the success rate of surgery was 100%. Assessment of equipment docking task load and intraoperative operating sensation score were similar between the two groups. Adverse events and Clavien–Dindo grade II or higher grade complication rates were comparable between the two groups. Device arm docking time, robotic arm operation time, and intraoperative bleeding were not significantly different between the two groups. Similar results were obtained from postoperative pathological outcomes and internal environment indexes.

**Conclusions:**

The efficacy and safety of the Kangduo Robotic Surgical System has been proved, operation of the Kangduo Robotic System by experienced surgeons for CC is not less effective than the da Vinci robotic System.

## Introduction

Highlights

- Operation of the Kangduo Robotic System by experienced surgeons for colon cancer is not less effective than the da Vinci robot.
- With proficiency in the da Vinci system, only eight kangduo robotic operations need to be done to become proficient with this system.
- Comparison of short-term outcomes of Robotic-assisted radical colon cancer surgery using the Kangduo Surgical Robotic system and the Da Vinci Si Robotic System–A prospective cohort study.

Colon cancer (CC) is currently one of the most common malignant tumors of the gastrointestinal system worldwide, with high morbidity and mortality rates^1^. CC radical surgery is currently the standard strategy for the treatment of nonmetastatic CC, and laparoscopic surgery is also recognized as the treatment of choice, which has the advantages of less surgical trauma, less bleeding, and shorter recovery time^2,3^. Although laparoscopic surgery is a more advantageous surgical platform, there are several drawbacks that limit its further development, including poorly defined field of view, demanding suturing techniques, relatively poor ergonomics, and rising operator fatigue^4^. However, the invention and introduction of robotic surgical systems have facilitated the further development of minimally invasive surgery^5^.

**Figure 1:**
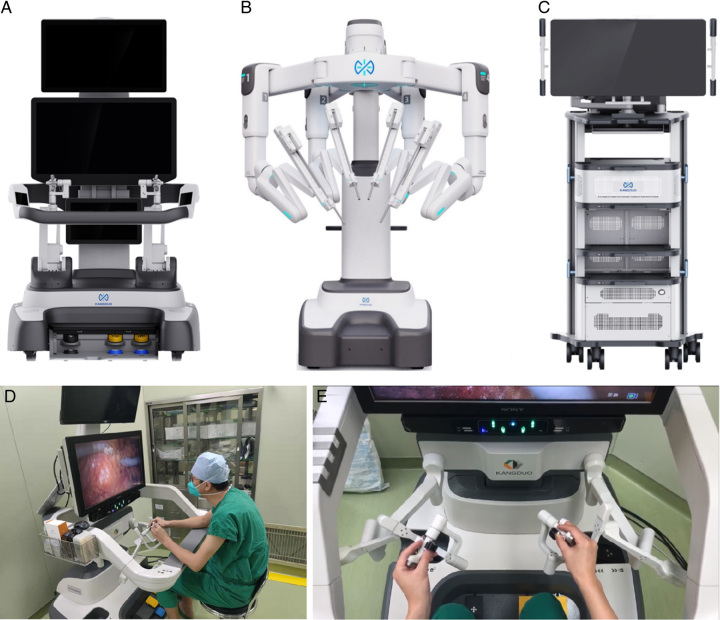
The KD surgical robot system: A. the surgeon console; B. the patient cart; c. the vision cart; D, E. a surgeon performs the operation using the KD-SR-01 system.

In 2000, the da Vinci robotic system (Intuitive Surgical, Inc.) was approved for clinical use in the U.S.A. and spread across the country because of its high-definition camera system and flexible instrument arms. This was due to Intuitive Surgical (ISRG), which launched a revolution in robotic surgery by providing the first superior robotic surgical system and global service centers. But the nature of technological development is to pursue innovation and diversity, and as regulations and requirements allow, this competitive operating platform is being developed all over the world, such as Revo-i in South Korea and Hinotori in Japan, and CMR: Versius in Europe. However, a new robotic platform technology developed in China, called the Kangduo surgical robot-01 (KD-SR-01, Kangduo Robot Ltd.) has been developed and consists of an open surgical console, three robotic arms, and a 3D video imaging system (Fig. 1). This robotic surgical platform technology has been scientifically reported in the field of urology^6,7^, but little has been reported in CC surgery. The purpose of this study is to compare the quality of surgery and short-term oncological outcomes of the KD-SR-01 (KD group) and the da Vinci Robotic System (DV group) in CC patients.

## Methods

### Study population

Between July 2022 and May 2023, 101 patients were assessed for eligibility. One patient was excluded prior to registration (not suitable for robotic surgery due to ASA grade III). Hundred colorectal cancer patients were enrolled and subjected to randomization, 42 patients with rectal cancer were excluded. There were 58 patients with CC, 28 in the KD group and 30 in the DV group. All surgeries were performed by three expert surgeons (Prof. Guiyu Wang from the Second Affiliated Hospital of Harbin Medical University, Prof. Zhengqiang Wei from the First Affiliated Hospital of Chongqing Medical University, and Prof. Yi Xiao from Peking Union Medical College Hospital, Chinese Academy of Medical Sciences and Peking Union Medical College).

Inclusion criteria were: 1) pathological biopsy confirmed colon adenocarcinoma; 2) age 18–80 years old; 3) no infiltration into surrounding organs and tissues and no metastasis on imaging; 4) ASA grades I–II; 5) voluntary participation in this trial and signing of informed consent. Exclusion criteria were: 1) patients with familial adenomatous polyposis and cancerous lesions of ulcerative colitis; 2) those who could not tolerate anesthesia and luminal surgery; 3) BMI >30 kg/m^2^; 4) combined with intestinal obstruction, hemorrhage, and perforation requiring emergency surgery; 5) Pregnant or lactating women; 6) Participants in other investigational drug or device clinical trials that were not completed. Withdrawal criteria were: 1) withdrawal of informed consent by the subject; 2) those who were deemed by the investigator to be no longer suitable for continuation of the clinical trial (present with worsening condition requiring emergency surgery, aggravation of underlying disease affecting surgery, and surgical exploration inconsistent with imaging examinations, etc.); 3) death of the subject; 4) loss of the subject to follow-up; and 5) request by the sponsor to terminate the trial.

The work has been reported in line with the strengthening the reporting of cohort, cross-sectional and case–control studies in surgery (STROCSS) criteria^8^ (Supplemental Digital Content 1, http://links.lww.com/JS9/B494). The study was approved by the institutional review board and ethics committee of each participating center. All patients provided written informed consent. The trial was conducted in accordance with the ethical principles of the Declaration of Helsinki and the Good Clinical Practice Guidelines. And the study is registered at www.chictr.org.cn (ChiCTR2200063172).

### Procedures

The participating surgeons are experienced and have all independently performed more than 100 robotic surgeries for colorectal cancer. Before participating in the trial, Kangduo Robotics ensured that the surgeons had sufficient time to learn and use the instruments.

**Figure 2:**
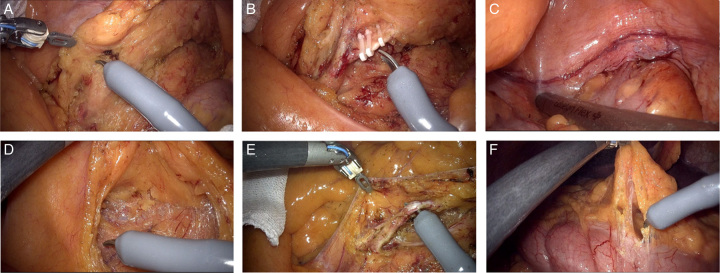
Procedure for left hemicolectomy. A. Lymphatic adipose tissue clearance; B. arterial clamping and dissection; C. distal transection of the tumor. Procedure for right hemicolectomy. D Separation of ileocolic vessels; E. Lymphatic fatty tissue clearance; F. Exposure of the right colon.

All patients underwent preoperative ultrasound, CT, or MRI, which was used to assess local tumor infiltration and preoperative staging. Candidate patients were grouped after a standard randomization procedure, with the KD group operated with the Kangduo robotic system and the DV group with the da Vinci robotic system. The operating procedures were similar for both. Perioperative management was performed according to the standards of each center, with no significant differences between the two groups. Detailed surgical procedures were performed according to the surgeon’s habits, but all were subject to the same principles. The upper surgical margin of the right half CC was in the ileum, 15 cm from the ileocecal space, and lower surgical margin was ≥10 cm from the tumor. The left half CC margins were all ≥10 cm from the tumor, for both upper and lower surgical margins. Tumor, mesentery, and regional lymph nodes were resected together. Radical surgery for CC is recommended to follow CME principles. All other intraoperative details were left to the discretion of the operator, such as whether to perform frozen section, whether to perform a diverting stoma, and whether to perform open conversion. All operative videos and photographs of the sample were preserved for postoperative review (Fig. 2).

Preoperative patients’ basic information and imaging information were recorded; intraoperative information such as operation time, device docking time, robotic arm operation time, blood loss, and blood transfusion were recorded; postoperative patients’ recovery was observed, and the main examination items included: blood index examination on the 1st day, the 3rd day, and the 4th week postoperatively; and postoperative complications were described by the Clavien–Dindo grade. A comprehensive evaluation of the safety and efficacy of the Kangduo Surgical Robotic System for laparoscopic resection of CC will be performed. The surgeon will also evaluate the maneuverability of the device in order to confirm the rationality of the product’s structural design and the ease of use of the supporting instruments.

### Outcomes

Efficacy evaluation: The primary efficacy index was the surgical success rate, which was defined as completing the robotic surgery as planned and not switching to another procedure. Secondary efficacy was the time to first flatus and the surgeon’s satisfaction with the operation of the robotic system: (1) assessment of equipment docking task load: using the NASA-TLX measurement scale; and (2) intraoperative operating sensation score.

Safety evaluation: operation time, blood loss and blood transfusion, occurrence of all adverse events. Device-related operation time: (1) Docking (Docking) time: moving the surgical arm system to the operating table to the last sheath docked to the corresponding surgical arm system machine arm. (2) Console time: the time from the first surgical operation to the completion of the operation.

### Statistical analysis

This is a noninferiority experiment and a total of 58 CC patients were collected from three experimental centers. The full analysis set (FAS), protocol program set(PPS), and safety set(SS) were consistent in this study. All the statistical analyses were calculated in statistical software package SPSS 22.0 (IBM Corp). Continuous variables were presented as mean±SD or median with the first to third quartile (Q1–Q3). Categorical data are shown as numbers with percentages. Comparisons of continuous variables were performed using the parametric test or Mann–Whitney test, and comparisons of categorical variables were performed using the χ^2^test or Fisher’s test. *P*<0.05 was considered statistically significant.

## Results

**Figure 3:**
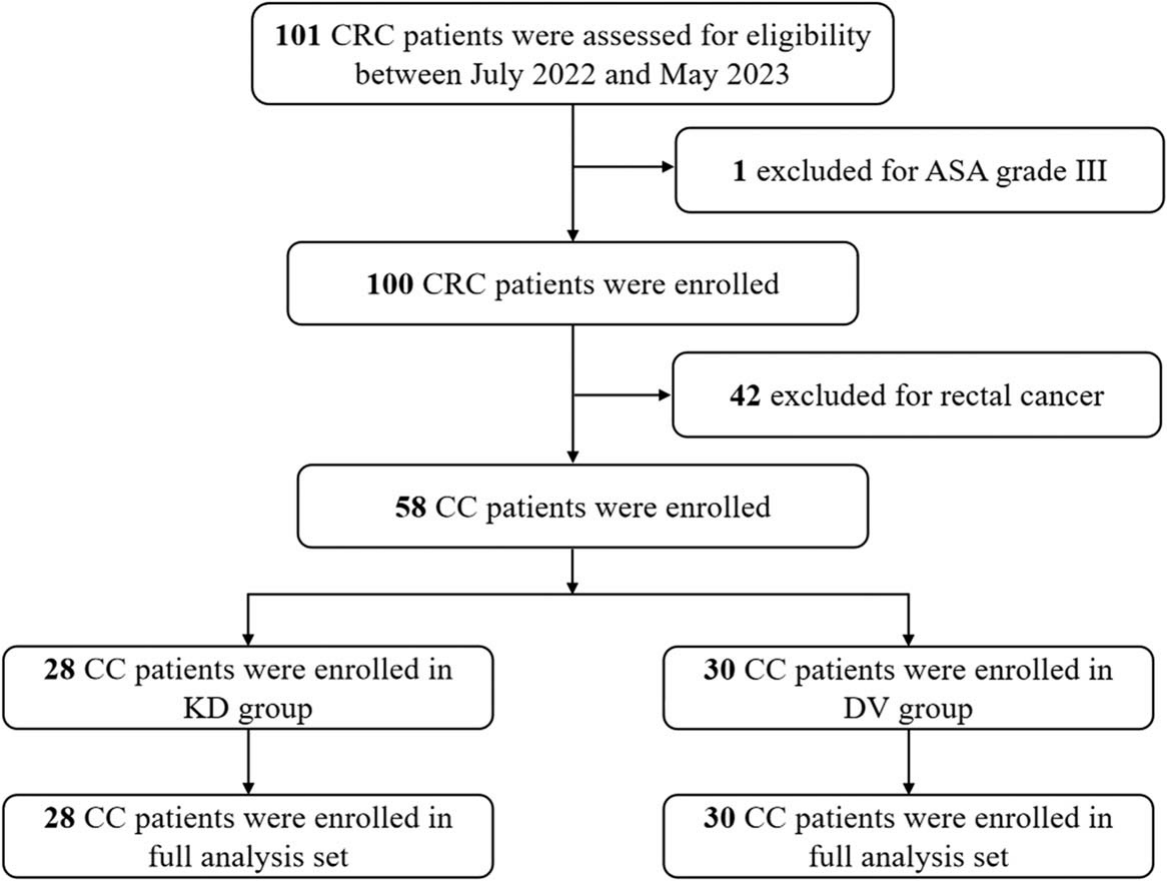
Trial profile.

A Trial profile is shown in Figure 3. The baseline characteristics of the patients in both groups were similar (Table 1). All three surgeons performed Kangduo robotic surgery and da Vinci robotic surgery. In the KD group, eight patients had tumor location in the right colon and 20 in the left colon. In the DV group, eight patients had tumor location in the right colon and 22 in the left colon. The overall Clavien–Dindo grade Ⅱ or higher grade or higher complication rate was 3.4%, the adverse event rate was 10.3%, and the serious adverse event rate was 6.9% (Table 2).

**Table 1:** Clinicopathological characteristics of enrolled patients.

Baseline characteristics	KD group (*n*=28)	DV group (*n*=30)	*P*
Sex (*n*,%)			0.882
Male	21 (75)	23 (76.7)	
Female	7 (25)	7 (23.3)	
Age, years (mean, SD)	62 (11)	61 (12)	0.711
BMI, kg/m^2^ (mean, SD)	24 (3)	24 (3)	0.478
ASN class (*n*, %)			1.000
I	2 (7.1)	2 (6.7)	
II	26 (92.9)	28 (93.3)	
Comorbidity (*n*, %)			0.115
Hypertension	11 (39.3)	10 (33.3)	
Diabetes	12 (42.9)	3 (10.0)	
Cardiovascular disease	3 (10.7)	2 (6.7)	
Surgical history (*n*, %)			0.096
No	20 (71.4)	15 (50)	
Yes	8 (28.6)	15 (50)	
Tumor location (*n*, %)			0.871
Right colon	8 (28.6)	8 (26.7)	
Left colon	20 (71.4)	22 (73.3)	
Pathological characteristics			
Tumor differentiation (*n*,%)			1.000
Well	3 (10.8)	4 (13.3)	
Moderate	21 (75.0)	22 (73.3)	
Poor	2 (7.1)	2 (6.7)	
Missing	2 (7.1)	2 (6.7)	
Pathological T stage (*n*, %)			0.793
T_1_	5 (17.9)	5 (16.7)	
T_2_	4 (14.2)	4 (13.3)	
T_3_	7 (25.0)	11 (36.7)	
T_4a_	12 (42.9)	10 (33.3)	
Pathological N stage (*n*, %)			0.700
N0	19 (67.9)	17 (56.6)	
N1	5 (17.9)	8 (26.7)	
N2	4 (14.2)	5 (16.7)	
TNM stage (*n*, %)			0.498
I	8 (28.6)	5 (16.7)	
II	11 (39.3)	11 (40.0)	
III	9 (32.1)	13 (43.3)	
Perineural invasion (*n*, %)	9 (32.1)	12 (40.0)	0.534
Lymphatic or vascular invasion (*n*, %)	8 (28.6)	8 (26.7)	0.876

**Table 2:** Surgical, pathological, and postoperative outcomes of enrolled patients.

	KD group (*n*=28)	DV group (*n*=30)	*P*
Success (*n*, %)	28 (100)	30 (100)	–
Conversions (*n*, %)	0 (0)	0 (0)	–
Duration of operation, min	200.7 (48.4)	164.4 (42.9)	0.004
Docking time, min	5.5 (2.0)	6.1 (3.4)	0.443
Console time, min	91.7 (50.2)	74.3 (37.3)	0.137
Blood loss, ml	58.9 (52.3)	54.7 (46.4)	0.744
Maximum tumor diameter, cm	3.9 (1.5)	4.4 (1.8)	0.288
Number of harvested lymph nodes	15.3 (7.9)	16.7 (6.8)	0.468
Negative margins (*n*, %)	56 (100)	60 (100)	–
Time to first flatus, h	31.3 (17.9)	33.4 (16.4)	0.640
Adverse events (*n*, %)	4 (14.3)	2 (6.7)	0.415
Serious adverse events (*n*, %)	2 (7.1)	2 (6.7)	1.000
Clavien–Dindo grade II or higher grade, (*n*, %)	1 (3.6)	1 (3.3)	1.000

### Efficacy of the device

The surgical success rate in the KD group and the DV group was 100%, with no statistical difference between the two groups. Time to first flatus was similar between the KD group and the DV group (31.3±17.9 h vs. 33.4±16.4 h, *P*=0.640) (Table 2). The differences between the KD group and the DV group were not statistically significant when comparing the scores for the assessment of the task load of equipment docking (NASA-TLX scale score) and the intraoperative operating sensation score (Table 3).

**Table 3:** NASA-TLX score.

	KD group (*n*=28)	DV group (*n*=30)	*P*
NASA-TLX			
Mental demand			0.342
Mean, SD	3.54 (3.42)	2.83 (2.04)	
Median,Q1–Q2	2.0 (1.0–4.75)	2.5 (1.0–4.0)	
Min, Max	1, 16	1, 9	
Physical demand			0.614
Mean, SD	2.79 (2.32)	2.53 (1.28)	
Median, Q1–Q2	2.0 (1.0–4.0)	2.5 (1.0–3.25)	
Max, Min	10, 0	1, 6	
Temporal demand			0.329
Mean, SD	3.36 (3.36)	2.67 (1.81)	
Median,Q1–Q2	2.0 (1–4.75)	2.0 (1.0–4.0)	
Max, Min	1, 15	1, 7	
Performance			0.362
Mean, SD	2.57 (2.67)	2.07 (1.14)	
Median, Q1–Q2	1.5 (1.0–3.0)	2.0 (1.0–3.0)	
Max, Min	1, 10	1, 5	
Effort			0.402
Mean, SD	3.14 (2.95)	2.60 (1.73)	
Median, Q1–Q2	2.0 (1.0–4.0)	2.0 (1–3.25)	
Max, Min	1, 10	1, 8	
Frustration			0.281
Mean, SD	2.68 (2.50)	2.13 (1.11)	
Median, Q1–Q2	2.0 (1.0–3.0)	2.0 (1.0–3.0)	
Max, Min	1, 9	1, 5	
Operation Feeling Score			1.000
well (*n*, %)	0	0	
moderate (*n*, %)	27 (96.4)	30 (100)	
poor (*n*, %)	1 (3.6)	0	
bad (*n*, %)	0	0	

### Safety of the device

Overall, the mean operative time was 200.7±48.4 min in the KD group and 164.4±42.9 min in the DV group, which was statistically different between the two groups (*P*=0.004). However, there was no statistical difference between the device docking time (*P*=0.443) and robotic arm operation time (*P*=0.137) between the two groups. There was also no statistically significant difference in the blood loss between the KD and DV groups (58.9±52.3 ml vs. 54.7±46.4 ml, *P*=0.744). No intraoperative complications occurred in either group (Table 2).

**Figure 4:**
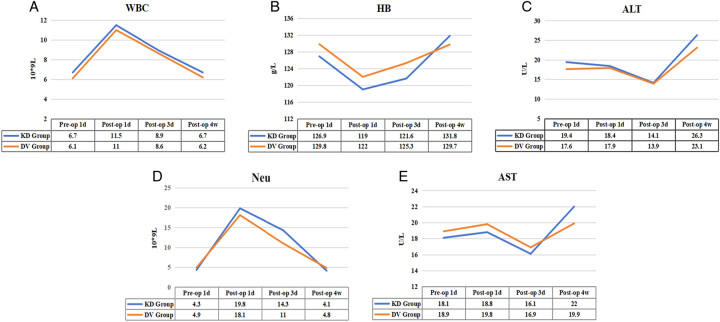
Dynamics of the average level of blood indexes. A. White blood cell (WBC); B. Hemoglobin(HB); C. Alanine transaminase (ALT); D. Neutrophils (Neu); E. Aspartate transaminase(AST).

Table 2 also demonstrates the occurrence of adverse events and Clavien–Dindo grade Ⅱ or higher grade in the patients, with a total of six surgery-related adverse events (three abdominal infections, one incisional infection, one incisional bleeding, and one Trocar hole bleeding), four surgery-related serious adverse events (three abdominal infections, one small bowel obstruction), and two Clavien–Dindo grade Ⅱ or higher grade. There were no significant differences between the two groups. After systematic treatment, the symptom disappeared and the patient was discharged from the hospital. An ultrasound examination 4 weeks after the operation showed no significant difference. The results of laboratory examinations showed no significant difference between the two groups in the changes of preoperative and postoperative blood indexes (Fig. 4 and Supplementary Table 1, Supplemental Digital Content 2, http://links.lww.com/JS9/B495).

Subgroup analysis showed (Table 4) that for patients undergoing right hemicolectomy, there was no statistical difference between the KD and DV groups in terms of operative time (224.0±37.7 min vs. 189.9±44.1 min, *P*=0.119), docking time and control time. Bleeding was similar in both groups. There were also no statistical differences in the rates of postoperative complications of Clavien–Dindo grade II or higher, adverse events and serious adverse events. There were no differences in pathological outcomes such as maximum tumor diameter, upper and lower margins, and number of harvested lymph nodes. For patients undergoing right hemicolectomy, the KD group had a longer operative time compared to the DV group (191.4±50.0 min vs. 155.6±39.5 min, *P*=0.013), but there was no statistically significant difference in the docking time (*P*=0.945) and control time (*P*=0.223). Bleeding was similar in both groups. There were also no statistical differences in the rates of postoperative complications of Clavien–Dindo grade II or higher, adverse events, serious adverse events, and pathological outcomes.

**Table 4:** Surgical, pathological, and postoperative of patients undergoing different operative procedures.

	KD group	DV group	*P*
Right colon	*n*=8	*n*=8	
Duration of operation, min	224.0 (37.7)	189.9 (44.1)	0.119
Docking time, min	5.6 (1.8)	7.9 (5.5)	0.298
Console time, min	113.2 (44.2)	97.1 (22.6)	0.375
Blood loss, ml	61.3 (25.3)	68.8 (57.9)	0.742
Maximum tumor diameter, cm	4.1 (1.9)	5.2 (2.4)	0.330
Number of harvested lymph nodes	17 (6.5)	15 (8.2)	0.598
Adverse events (*n*, %)	1	2	1.000
Serious adverse events (*n*, %)	0	2	0.467
Complication of C-D II or higher, *n*	0	1	1.000
Left colon	*n*=20	*n*=22	
Duration of operation, min	191.4 (50.0)	155.6 (39.5)	0.013
Docking time, min	5.5 (2.1)	5.4 (2.0)	0.945
Console time, min	83.1 (51.0)	66 (38.4)	0.223
Blood loss, ml	58.0 (60.4)	49.6 (41.9)	0.598
Maximum tumor diameter, cm	3.8 (1.3)	4.0 (1.4)	0.560
Number of harvested lymph nodes	14.7 (8.4)	17.4 (6.4)	0.242
Adverse events (*n*, %)	3	0	0.099
Serious adverse events (*n*, %)	2	0	0.221
Complication of C-D II or higher, *n*	1	0	0.476

## Discussion

With the rapid development of minimally invasive concepts, surgeons are no longer satisfied with the benefits offered by traditional laparoscopic techniques and strive for an approach that allows patients to benefit more from minimally invasive procedures. The emergence of robotic surgical platforms has changed this^9^. Robotic surgical platforms can improve on many of the shortcomings of traditional laparoscopic techniques. The magnified 3D vision can help surgeons perform a clearer view of the surgical area, the three free moving arms can perform difficult surgical operations and it also frees up assistants. The feasibility, safety, and efficacy of robotic surgery have been proved in previous studies. An RCT trial from China reported the benefits of robotic surgery in improving oncological quality of resection for middle and low rectal cancer compared with conventional laparoscopic surgery^10^. Similar results were obtained in other areas^11^. The da Vinci robot is currently the most widely used surgical robot in the world. Incomplete statistics show that there are about 3000 da Vinci surgical robot around the world, while China is only equipped with about 100 units. Due to its high cost, which limits the development of this technology, it is extremely important to develop new robotic surgical platforms. The nature of technological development is the pursuit of innovation and diversity, and the Kangduo robotic is a surgical robotic system developed in China with its own intellectual property rights. In this randomized controlled trial, we demonstrated that the overall efficacy of the Kangduo robotic system is no worse than that of the da Vinci system in radical CC surgery. The results are encouraging in terms of both efficacy and safety of the Kangduo robotic system.

First, the efficacy of the Kangduo robotic system was assessed from one primary efficacy index and two secondary efficacy indexes. In this study, both the KD group and the DV group completed the surgery according to the established protocol and neither of them was transferred to conventional laparoscopic or open surgery, and the success rate of the surgery were 100%, with the KD group not inferior to the DV group. The time to first postoperative flatus was also comparable, and both groups were effective in protecting gastrointestinal function. There was no significant difference in intraoperative scores provided by the surgeons.

The emergence and development of a new operation technique must be based on a certain degree of safety. Radical surgery for CC is a relatively complex procedure that involves the freeing of the tumor, reconstruction of the gastrointestinal tract, and a variety of vascular system variants. The principles of sterile and tumor free are followed while completing tumor removal. For fat and strong patients, the narrow space in the abdominal cavity does nothing but add to the difficulty of the operation. Additionally, the medical center must have an experienced assistant; however, not always available. Together, these factors affect the quality of the operation, leading to failure, unexpected trauma, and tumor remains.

The Kangduo robotic system offers similar advantages to the da Vinci, with a robotic arm that can move freely in multiple angles, and is flexible and delicate. The many advantages expand the indications for CC surgery and allow surgeons to operate in a more complex abdominal setting^12^.

The results of this study showed that the total operative time in the KD group was significantly longer than that in the DV group; however, the machine arm operation time was relatively similar between the two groups, and we considered that the long total operative time in the KD group might be due to the confusion between the anesthesia and abdominal closure times. The results of a multicenter, triple-blind, randomized controlled trial^13^ showed that the mean operative time for robot-assisted right hemicolectomy was 146 min, which was different from our trial, but our between-group analysis indicated no significant difference. In addition, the results of a systematic review and meta-analysis^14^ indicated that the mean time for robot-assisted left hemicolectomy was 215.0 min, which was consistent with our study. Most importantly, oncologic outcomes also showed no significant differences between the two groups. This suggests that the quality of tumors resected with the Kangduo robotic system is not inferior to that of the da Vinci system.

Postoperative recovery of the patient is also a major concern for the surgeon, provided that the surgery is performed successfully. First, during our study period, all patients were successfully discharged from the hospital about 1 week after surgery, with no secondary surgeries and no deaths within 1 month. In this article, we analyzed only the occurrence of Clavien–Dindo grade Ⅱ or higher grade, as grade 1 complications were more common. The complication rate in our study was relatively low compared to previous studies^12–15^, which may be related to the surgeon’s skill level. In addition, laboratory examinations at 1, 3, and 5 days postoperatively were analyzed in this study with the aim of exploring whether there were any differences in the effects of robotic surgery on the internal environment in the body between the two groups. The results of laboratory examinations also showed similar results between the two groups. Overall, the short-term prognosis results derived from this study were favorable, although long-term prognosis was not a predefined endpoint of this study, and we will follow-up to provide long-term follow-up results.

**Figure 5:**
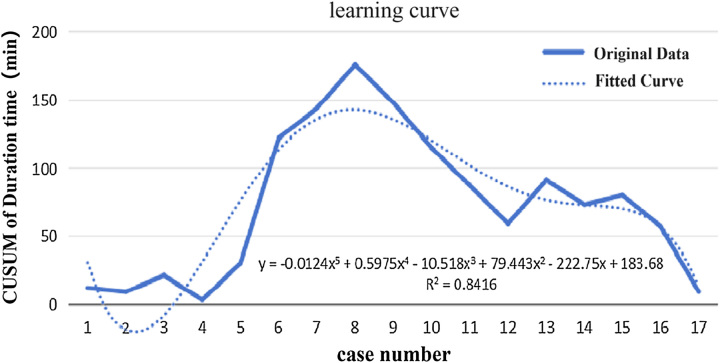
The learning curve of the duration time in center 2.

In addition, we found an exciting phenomenon in center 2, we plotted the learning curve of surgeon using the kangduo robotic system, and we found that the threshold was at the 8th patient, suggesting that with proficiency in the da Vinci system, only eight kangduo robotic operations need to be done to become proficient with this system. However, this is only a result obtained in a single center, and subsequent validation in a multicenter cohort is needed (Fig. 5).

It is understood that the Kangduo robotic system in has been validated in the animal experiment stage^16^. In addition, favorable results have been reported in urology clinical trials^6,17–19^. The strength of this study is the multicenter, prospective data collection and strict follow-up, which is highly persuasive. Our study also fills a relevant gap in colorectal cancer operation, which pushes the approval of the Kangduo robotic surgery system in the Chinese market, and then rapidly enters the international market, which is necessary to reduce the outcomes of the existing robotic surgical systems and benefit more patients, even though the price has not been determined yet. In addition, there are some limitations to this study, firstly, although the short-term operation results are encouraging, the long-term results are not yet known, and we will continue to follow-up in the future. Secondly, the three centers in this study were not fully standardized in terms of perioperative period as this is more difficult. Third, the dataset included in this study is a subcohort (CC cohort) of this noninferiority trial, although our results are positive and we hope to continue to improve and enhance the trial in the follow-up. In addition, the first-generation Kangduo robot also has certain shortcomings, such as not achieving the naked eye 3D effect, need to wear 3D glasses and no force feedback. We hope that we can continue to improve the performance of the Kangduo robot in the future.

## Conclusion

Operation of the Kangduo Robotic System by experienced surgeons for CC is not less effective than the da Vinci robot.

## Ethical approval

This study was initiated by Peking Union Medical College Hospital. Ethical review board approval was obtained and informed consent was obtained from the institutional review board (KS2022261).

## Consent for publication

Written informed consent was obtained from the patient for publication of this case report and accompanying images. A copy of the written consent is available for review by the Editor-in-Chief of this journal on request.

## Sources of funding

National Natural Science Foundation of China (62276084).

## Author contribution

Y.L.: formal analysis, methodology, software, writing – original draft; Y.W.: writing – original draft, and validation; C.W., X.Z., X.W., Y.Y., Z.W.: conceptualization, investigation, and resources; Y.X.: data curation, project administration, supervision, and visualization; G.W.: formal analysis, funding acquisition, methodology, writing – review and editing.

## Conflicts of interest disclosure

The authors declare that they have no known competing financial interests or personal relationships that could have appeared to influence the work reported in this paper.

## Research registration unique identifying number (UIN)

The trial was conducted in accordance with the ethical principles of the Declaration of Helsinki and the Good Clinical Practice Guidelines. And the study is registered at www.chictr.org.cn (ChiCTR2200063172). http://www.chictr.org.cn/index.aspx.

## Guarantor

Yi Xiao, Zhengqiang Wei, and Guiyu Wang.

## Data availability statement

The data which was used and/or analyzed during this study is available from the corresponding author on reasonable request.

## Provenance and peer review

Not commissioned, externally peer-reviewed.

## Supplementary Material

SUPPLEMENTARY MATERIAL
