# Supplementary material for: Comparison of short-term outcomes of robotic-assisted radical colon cancer surgery using the Kangduo Surgical Robotic System and the Da Vinci Si Robotic System: a prospective cohort study
Source: Int J Surg. 2023 Dec 4;110(3):1511–8. doi: 10.1097/JS9.0000000000000976 (PMC10942201; doi:10.1097/JS9.0000000000000976)
Supplement: SUPPLEMENTARY MATERIAL [file js9-110-1511-s002.docx]

**Supplementary table 1.The results of laboratory examinations**

|  | KD Group（n=28） | DV Group（n=30） | P value |
| --- | --- | --- | --- |
| AST on the preoperative day(n,%) |  |  | 0.175 |
| Normal | 25(89.3) | 22(73.3) |  |
| Abnormal | 2(7.1) | 7(23.3) |  |
| Missing | 1(3.6) | 1(3.4) |  |
| ALT on the preoperative day(n,%) |  |  | 1.000 |
| Normal | 25(89.3) | 27(90) |  |
| Abnormal | 3(10.7) | 3(10) |  |
| WBC on the preoperative day(n,%) |  |  | 0.665 |
| Normal | 25(89.3) | 28(93.3) |  |
| Abnormal | 3(10.7) | 2(6.7) |  |
| Neu on the preoperative day(n,%) |  |  | 1.000 |
| Normal | 26(92.9) | 27(90) |  |
| Abnormal | 2(7.1) | 3(10) |  |
| HB on the preoperative day |  |  | 0.457 |
| Normal | 17(60.7) | 21(70) |  |
| Abnormal | 11(39.3) | 9(30) |  |
| AST on the 1st postoperative day(n,%) |  |  | 0.380 |
| Normal | 25(89.3) | 23(76.7) |  |
| Abnormal | 3(10.7) | 6(20) |  |
| Missing | 0 | 1(3.3) |  |
| ALT on the 1st postoperative day(n,%) |  |  | 1.000 |
| Normal | 24(85.7) | 25(83.3) |  |
| Abnormal | 4(14.3) | 5(16.7) |  |
| WBC on the 1st postoperative day(n,%) |  |  | 0.486 |
| Normal | 7(25) | 10(33.3) |  |
| Abnormal | 21(75) | 20(66.7) |  |
| Neu on the 1st postoperative day(n,%) |  |  | 0.070 |
| Normal | 3(10.7) | 9(30) |  |
| Abnormal | 25(89.3) | 21(70) |  |
| HB on the 1st postoperative day(n,%) |  |  | 0.553 |
| Normal | 18(64.3) | 17(56.7) |  |
| Abnormal | 10(35.7) | 13(43.3) |  |
| AST on the 3rd postoperative day (n,%) |  |  | 0.280 |
| Normal | 15(53.6) | 20(66.7) |  |
| Abnormal | 13(46.4) | 9(30) |  |
| Missing | 0 | 1(3.3) |  |
| ALT on the 3rd postoperative day(n,%) |  |  | 0.380 |
| Normal | 24(85.7) | 23(76.7) |  |
| Abnormal | 4(14.3) | 7(23.3) |  |
| WBC on the 3rd postoperative day(n,%) |  |  | 0.649 |
| Normal | 20(71.4) | 23(76.7) |  |
| Abnormal | 8(28.6) | 7(23.3) |  |
| Neu on the 3rd postoperative day(n,%) |  |  | 0.309 |
| Normal | 16(57.1) | 21(70) |  |
| Abnormal | 12(42.9) | 9(30) |  |
| HB on the 3rd postoperative day(n,%) |  |  | 0.695 |
| Normal | 20(71.4) | 20(66.7) |  |
| Abnormal | 8(28.6) | 10(33.3) |  |
| AST on the 4th postoperative week (n,%) |  |  | 0.848 |
| Normal | 24(85.7) | 26(86.7) |  |
| Abnormal | 4(14.3) | 3(10) |  |
| Missing | 0 | 1(3.3) |  |
| ALT on the 4th postoperative week(n,%) |  |  | 0.344 |
| Normal | 25(89.3) | 28(93.4) |  |
| Abnormal | 3(10.7) | 1(3.3) |  |
| Missing | 0 | 1(3.3) |  |
| WBC on the 4th postoperative week(n,%) |  |  | 0.344 |
| Normal | 25(89.3) | 28(93.4) |  |
| Abnormal | 3(10.7) | 1(3.3) |  |
| Missing | 0 | 1(3.3) |  |
| Neu on the 4th postoperative week(n,%) |  |  | 1.000 |
| Normal | 27(96.4) | 27(90) |  |
| Abnormal | 1(3.6) | 2(6.7) |  |
| Missing | 0 | 1(3.3) |  |
| HB on the 4th postoperative week(n,%) |  |  | 1.000 |
| Normal | 20(71.4) | 21(70) |  |
| Abnormal | 8(28.6) | 8(26.7) |  |
| Missing | 0 | 1(3.3) |  |
